# Integrated Transcriptomic and Spatial Analyses Associate M2-like Myeloid Signatures with Neuroimmune Remodeling in Alzheimer’s Disease

**DOI:** 10.3390/ijms27104430

**Published:** 2026-05-15

**Authors:** Sz-Bo Wang, Kuan-Nien Chou, Yi-Lin Chiu

**Affiliations:** 1 School of Medicine, National Defense Medical University, Taipei 11490, Taiwan; 409010129@mail.ndmutsgh.edu.tw; 2 Taipei Veterans General Hospital, Taipei 11217, Taiwan; 3 Department of Neurological Surgery, Tri-Service General Hospital, National Defense Medical University, Taipei 11490, Taiwan; 4 Graduate Institute of Biochemistry, College of Biomedical Science, National Defense Medical University, Taipei 11490, Taiwan

**Keywords:** Alzheimer’s disease, M2-like myeloid signatures, immune deconvolution, spatial transcriptomics, single-cell RNA-seq, neuroinflammation

## Abstract

Alzheimer’s disease (AD) is characterized by progressive neurodegeneration and prominent neuroimmune remodeling, but the contribution of macrophage and myeloid states across disease severity remains incompletely defined. We integrated bulk transcriptomic, single-cell RNA sequencing (RNA-seq), and spatial transcriptomic datasets to characterize AD-associated myeloid immune changes across Braak stage and disease status. Across datasets, M2-like macrophage and myeloid signatures showed progressive enrichment with increasing neuropathological severity and were accompanied by pathway changes related to macrophage proliferation, TGF-β signaling, and myeloid homeostasis. Immune-feature-based classifiers identified macrophage-related variables among the informative features distinguishing AD from controls. CellChat analyses further inferred that M2-like myeloid populations occupied communication-enriched positions in single-cell and spatial interaction networks, including apolipoprotein E (ApoE), CX3C chemokine signaling, and fibronectin 1 (*FN1*)-associated signaling contexts. Collectively, these findings indicate that M2-like myeloid programs are consistently associated with AD severity and neuroimmune network remodeling. Rather than establishing a causal disease driver, this study highlights M2-like myeloid signatures as candidate neuroimmune components that warrant experimental validation in human-relevant systems.

## 1. Introduction

Alzheimer’s disease (AD) is a progressive neurodegenerative disorder, predominantly recognized by the deposition of β-amyloid plaques and neurofibrillary tangles containing tau proteins, essential for clinical diagnosis [1,2]. AD presents primarily in two distinct forms: familial early-onset and sporadic late-onset. Familial early-onset AD is associated with genetic mutations in amyloid precursor protein (*APP*), Presenilin 1 (*PSEN1*), and Presenilin 2 (*PSEN2*) genes. Sporadic late-onset AD, which accounts for the majority of cases, is closely linked to variations in the *APOE* gene, significantly influencing disease susceptibility and progression [3,4,5]. Genome-wide studies have also identified additional genes involved in immune responses, lipid metabolism, and protein clearance mechanisms, suggesting a multifactorial genetic landscape contributing to AD’s pathogenesis [6,7].

Age remains the primary risk factor, with a notable increase in incidence after age 85. However, the interplay of genetic background, sex, and environmental factors significantly modulates both the risk and timing of disease onset [8,9]. Current therapeutic strategies predominantly target amyloid and tau pathways due to their central roles in plaque and tangle formation. Emerging treatments focus on kinase inhibitors to prevent tau phosphorylation and aggregation, alongside antioxidants and estrogen compounds hypothesized to confer neuroprotection against oxidative stress-induced neuronal damage [10].

The amyloid hypothesis posits that disrupted production or clearance of amyloid β-protein (Aβ) initiates a cascade of neurotoxic events, leading to plaque deposition and neuronal dysfunction, marking the onset of AD pathology [11,12,13,14]. Concurrently, tau protein undergoes abnormal posttranslational modifications, primarily hyperphosphorylation, resulting in the formation of neurofibrillary tangles. These tangles are directly implicated in neuronal death, contributing significantly to the cognitive decline observed in AD patients [13,15,16,17,18].

Increasingly, neuroinflammation is recognized as a critical driver in AD pathogenesis [19]. Activated microglia and astrocytes release pro-inflammatory mediators, exacerbating neuronal injury and accelerating disease progression [13,20]. Microglia, particularly bone marrow-derived subsets, exhibit complex roles, including limiting amyloid plaque formation and modulating disease trajectory [21,22]. Genetic factors, notably *APOE* and *TREM2*, profoundly influence microglial activation and immune responses, thereby affecting AD progression [23,24]. A specialized subset, known as disease-associated microglia (DAM), is identified in AD contexts and may provide protective effects against neurodegeneration, highlighting their potential as therapeutic targets [25]. Additionally, peripheral immune cells such as monocytes and lymphocytes actively interact with central nervous system (CNS) immunity, further influencing the disease course and presenting additional therapeutic avenues [26].

Moreover, microglial dysfunction characterized by altered cytokine profiles and disrupted signaling pathways often precedes overt pathological features, indicating early inflammatory involvement in disease initiation [27,28]. Despite advancements, a significant knowledge gap persists regarding the role of peripheral myeloid populations, particularly alternatively activated (M2-like) macrophages, in the AD neuroimmune environment. Emerging evidence from transcriptomic and histopathological analyses suggests these macrophages infiltrate aging brains, adopting context-dependent phenotypes that can either exacerbate or ameliorate neurodegeneration. However, existing studies frequently lack spatial resolution and comprehensive analysis, complicating definitive interpretations regarding their functional roles.

Therefore, this study aimed to characterize AD-associated macrophage and myeloid remodeling by integrating bulk transcriptomic, single-cell RNA sequencing (RNA-seq), and spatial transcriptomic analyses. Specifically, we sought to (i) determine whether macrophage and M2-like myeloid signatures change with Braak stage and AD status; (ii) identify pathway modules associated with these immune alterations; (iii) evaluate whether immune-cell profiles can distinguish AD from control samples; and (iv) infer cell-cell communication patterns involving M2-like myeloid populations in single-cell and spatial contexts. Given the exploratory and computational nature of these analyses, our objective was to generate testable hypotheses regarding AD-associated neuroimmune remodeling rather than to establish causal cellular hierarchies.

## 2. Results

### 2.1. Gene Set Variation Analysis (GSVA) Reveals Distinct Pathway Enrichment Patterns Across Braak Stages in Alzheimer’s Disease

Gene Set Variation Analysis (GSVA) was performed on each sample utilizing hallmark gene sets and curated pathways from Molecular Signatures Database (MSigDB), encompassing C2 (KEGG, Reactome, WikiPathways), C5 (Gene Ontology: Biological Process, Molecular Function, Cellular Component), C7 (immune signatures), and supplementary pathways designated as “Others”. The analysis revealed significant alterations in GSVA scores corresponding to advancing Braak stages, thereby delineating distinct molecular profiles among healthy control, preclinical Alzheimer’s disease (pre-AD), and Alzheimer’s disease (AD) cohorts. Pearson correlation coefficients were computed between the GSVA enrichment scores and Braak stages. Pathways demonstrating significant correlations (*p*-value < 0.05) were identified, with the top five positively and negatively correlated gene sets for each database being highlighted. This correlational analysis underscored notable associations with age-related neuroinflammation and macrophage activation.

Prominent pathways identified included “GSE5099 UNSTIM VS MCSF TREATED MONOCYTE DAY3 UP,” indicating the involvement of Macrophage Colony-Stimulating Factor (MCSF) in macrophage survival, differentiation, and M2 polarization. The “GSE360 L DONOVANI VS T GONDII MAC UP” pathway reflected macrophage activation in response to pathogenic stimuli and its impact on the M1/M2 polarization balance. Furthermore, the “GSE7348 UNSTIM VS LPS STIM MACROPHAGE UP” pathway, associated with lipopolysaccharide (LPS)-stimulated macrophages, pointed to immune activation and neuroinflammatory cascades.

**Figure 1:**
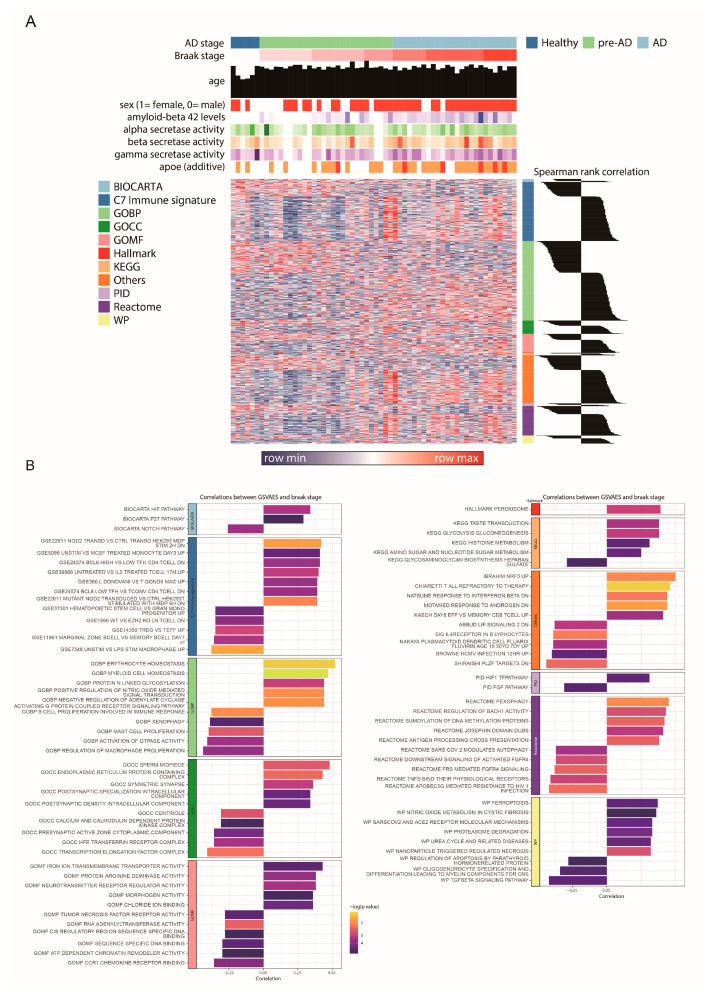
Gene Set Variation Analysis (GSVA)-based Pathway Activity and Correlation Patterns Across Braak Stages in Alzheimer’s Disease. (**A**) Heatmap illustrating GSVA scores for significantly altered pathways from MSigDB hallmark, C2, C5, C7, and other curated gene sets. Samples are annotated by Alzheimer’s disease (AD) stage (Healthy, pre-AD, AD), Braak stage, age, sex, Aβ1–42 levels, tau levels, and secretase activities. Rows represent individual pathways, colored by their database of origin. Spearman rank correlation plots on the right depict the correlation of pathway enrichment scores with Braak stage. (**B**) Bar plots showcasing the top five positively and negatively correlated pathways with Braak stage for each database, filtered by a *p*-value < 0.05. Bars are colored by their correlation coefficient (purple for positive, yellow for negative), and the x-axis represents the −log10 (*p*-value).

Within the Gene Ontology Biological Process (GOBP) category, pathways such as “MYELOID CELL HOMEOSTASIS” were implicated, relating to the integrity and functionality of myeloid cells, including macrophages. “POSITIVE REGULATION OF NITRIC OXIDE MEDIATED SIGNAL TRANSDUCTION” highlighted the contribution of nitric oxide to macrophage activation and neuroinflammation. Additionally, “REGULATION OF MACROPHAGE PROLIFERATION” was closely associated with macrophage biology and M2 polarization. In the Gene Ontology Molecular Function (GOMF) category, “NEUROTRANSMITTER RECEPTOR REGULATOR ACTIVITY” was found to influence neuronal signaling and microglial activation, while “TUMOR NECROSIS FACTOR RECEPTOR ACTIVITY” connected to immune responses integral to neuroinflammation. Other noteworthy pathways included “ANTIGEN PROCESSING CROSS PRESENTATION” from Reactome, pertinent to macrophage activation, and “TNFS BIND THEIR PHYSIOLOGICAL RECEPTORS,” emphasizing *TNF* signaling in immune activation. The WikiPathways “WP FERROPTOSIS” explored ferroptotic cell death pathways influenced by macrophage activity, and “WP TGFBETA SIGNALING PATHWAY” underscored the regulatory role of Transforming Growth Factor-beta (TGF-β) in immune responses and M2 macrophage polarization. Collectively, these analyses elucidate nuanced patterns of pathway activity across different Braak stages, offering insights into the molecular mechanisms underpinning the progression of Alzheimer’s disease. These GSVA-based pathway activity and Braak-stage correlation patterns are summarized in Figure 1.

### 2.2. Weighted Gene Co-Expression Network Analysis Identifies Gene Modules and Pathways Correlated with Alzheimer’s Disease Neuropathology

**Figure 2:**
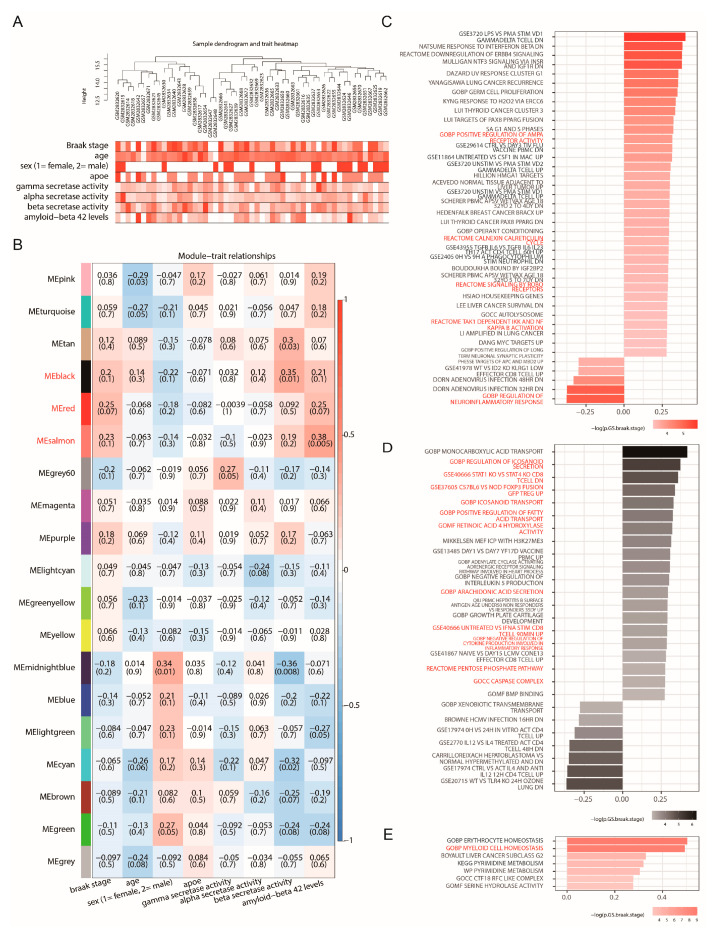
WGCNA Reveals Co-expression Modules and Enriched Pathways Associated with Braak Stage in Alzheimer’s Disease. (**A**) Heatmap of ME values for WGCNA-identified co-expression modules (rows, labeled by module color) across patient samples (columns). The dendrogram at the top illustrates hierarchical clustering of patient samples based on their ME profiles. Orange-red colors denote higher ME values, while white indicates lower values. (**B**) Heatmap depicting the correlation between ME (rows identified by colors) from WGCNA and clinical traits (columns), with a primary focus on Braak stage. Red cells indicate positive correlations, and blue cells represent negative correlations. Numbers within cells correspond to the correlation coefficient and the *p*-value (in parentheses). Modules MEred, MEsalmon, and MEblack demonstrate the most significant correlations with Braak stage. (**C**–**E**) Bar plots illustrating the correlation of significantly enriched biological process gene sets within the (**C**) MEred, (**D**) MEblack, and (**E**) MEsalmon modules with Braak stage. In panels (**C**–**E**), pathway labels shown in red indicate gene sets highlighted for their relevance to Alzheimer’s disease-related neuroinflammation, macrophage/M2-like myeloid biology, microglial activation, or broader immune-cell functions.

WGCNA was employed to identify gene expression patterns associated with AD neuropathology. As an initial step in understanding sample relationships based on gene expression, ME values, representing the primary expression profile of each identified co-expression module, were visualized. Figure 2A presents a heatmap of these ME values (rows, indicated by module colors) across all patient samples (columns). A hierarchical clustering dendrogram is displayed above the heatmap, illustrating the grouping of patient samples based on their overall ME profiles, with orange-red colors indicating higher ME values and white indicating lower values. This allowed for the visualization of sample clusters exhibiting similar module activity patterns. The WGCNA methodology proceeded by constructing a scale-free gene co-expression network. Pairwise gene correlations were used to generate a similarity matrix, which was transformed into an adjacency matrix and subsequently into a topological overlap matrix (TOM). Hierarchical clustering applied to the TOM grouped genes with concordant expression patterns into distinct modules, each assigned a unique color identifier. The MEs were then correlated with clinical traits, particularly Braak stage, a key indicator of AD neurofibrillary tangle pathology. The heatmap in Figure 2B illustrates the strength of these correlations, with red indicating positive and blue indicating negative correlations between MEs (rows) and clinical traits (columns). Notably, modules designated MErED, MEsalmon, and MEblack exhibited the strongest correlations with Braak stage, highlighting their potential significance in AD pathogenesis.

Further investigation focused on the biological implications of these key modules. The correlation and statistical significance of biological process gene sets within MEred, MEsalmon, and MEblack with Braak stage are depicted in Figure 2C–E, respectively. In these plots, the length of each bar corresponds to the magnitude of the correlation coefficient, while the color intensity signifies statistical significance. Pathway analysis of these three modules revealed a significant enrichment in 15 pathways related to neuroinflammation, Alzheimer’s disease mechanisms, macrophage M2 polarization, microglial activation, and broader immune cell functions. The MEred module (Figure 2C) demonstrated upregulation of the “REACTOME TAK1 DEPENDENT IKK AND NF KAPPA B ACTIVATION” pathway, critical for initiating inflammatory responses in microglia. Also upregulated were “GOBP POSITIVE REGULATION OF AMPA RECEPTOR ACTIVITY,” “REACTOME CALNEXIN CALRETICULIN CYCLE,” and “REACTOME SIGNALING BY ROBO RECEPTORS.” In contrast, the “GOBP REGULATION OF NEUROINFLAMMATORY RESPONSE” pathway was downregulated. The MEblack module (Figure 2D) showed upregulation of several pathways, including “GOBP NEGATIVE REGULATION OF CYTOKINE PRODUCTION INVOLVED IN INFLAMMATORY RESPONSE,” “GSE40666 UNTREATED VS IFNA STIM CD8 TCELL 90MIN UP,” “GOBP REGULATION OF ICOSANOID SECRETION,” “GOBP ICOSANOID TRANSPORT,” “GOBP POSITIVE REGULATION OF FATTY ACID TRANSPORT,” “GOBP ARACHIDONIC ACID SECRETION,” “GOMF RETINOIC ACID 4 HYDROXYLASE ACTIVITY,” “REACTOME PENTOSE PHOSPHATE PATHWAY,” “GOCC CASPASE COMPLEX,” “GSE40666 STAT1 KO VS STAT4 KO CD8 TCELL DN,” and “GSE37605 C57BL6 VS NOD FOXP3 FUSION GFP TREG UP.” These findings suggest multifaceted roles in immune regulation, lipid metabolism, oxidative stress, and apoptosis within the CNS. The MEsalmon module (Figure 2E) was characterized by the upregulation of “GOBP MYELOID CELL HOMEOSTASIS,” a pathway essential for maintaining the balance and functionality of microglia and macrophages.

### 2.3. Progressive Immune Activation, Shifting Cellular Landscapes, and Neuronal Loss Characterize Advancing Neuropathology in Alzheimer’s Disease

**Figure 3:**
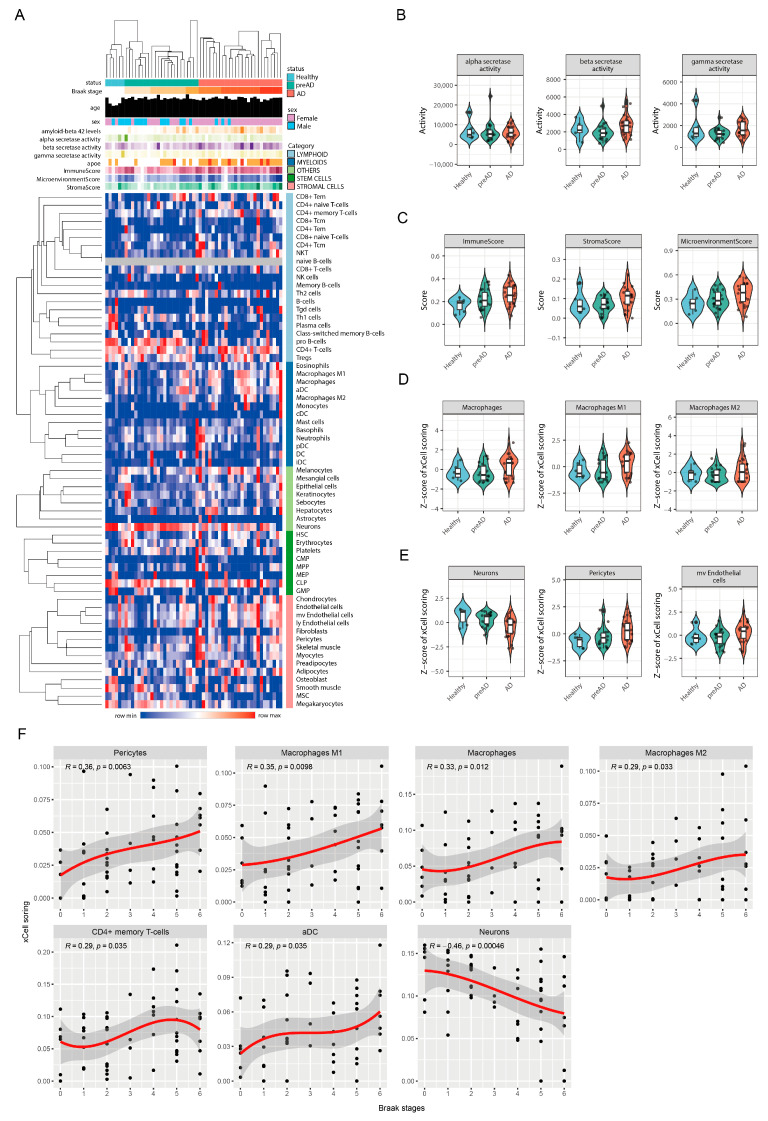
xCell Analysis Reveals Altered Immune Cell Infiltration and Cellular Changes Correlated with Alzheimer’s Disease Progression and Braak Stage. (**A**) Heatmap displaying immune cell infiltration status assessed by xCell based on gene expression data. Samples (columns) are hierarchically clustered based on similarities in inferred immune cell expression profiles and annotated at the top with AD stage, Braak stage, age, sex, Aβ1–42 levels, tau levels, and secretase activities. Rows represent different cell types, clustered by similarity in their profiles across samples. Red indicates higher inferred cell type abundance (xCell scores), while blue indicates lower abundance. (**B**) Violin plots comparing the activity levels of alpha-secretase, beta-secretase, and gamma-secretase across healthy control (Healthy), preclinical AD (pre-AD), and Alzheimer’s disease (AD) patient groups. (**C**) Violin plots illustrating the Immune score, Stroma score, and Microenvironment score across Healthy, pre-AD, and AD groups. (**D**) Violin plots presenting the Z-scores of xCell enrichment scores for total macrophages, M1 macrophages, and M2 macrophages in Healthy, pre-AD, and AD groups. (**E**) Violin plots depicting the Z-scores of xCell enrichment scores for neurons, pericytes, and microvascular endothelial (mv Endothelial) cells in Healthy, pre-AD, and AD groups. (**F**) Scatter plots showing the correlation between xCell enrichment scores of various immune and stromal cell types and Braak stages.

To investigate the immune cell infiltration landscape associated with AD progression, xCell analysis was performed on gene expression data. This analysis considered clinical and biochemical variables including age, amyloid-beta 42 levels, alpha-, beta-, and gamma-secretase activities, *APOE* status, sex, as well as calculated Immune, Microenvironment, and Stroma scores. The expression levels of various cell types, including lymphoid, myeloid, stem, and stromal cells, were quantified. A heatmap illustrating these xCell scores (Figure 3A) demonstrated hierarchical clustering of samples based on their immune cell profiles, with red indicating higher and blue indicating lower inferred cell type abundance. Pearson correlation coefficients and *p*-values for the association between cell type scores and Braak stage, displayed on the right of the heatmap, highlighted significant relationships. The activity levels of alpha-, beta-, and gamma-secretases were found to exhibit minimal differences across healthy control, preclinical AD (pre-AD), and AD patient groups, suggesting relative stability of these enzymatic activities during the observed stages of AD progression (Figure 3B). In contrast, the Immune score, Stroma score, and Microenvironment score all demonstrated an upward trend from healthy controls to AD patients, indicative of progressive immune cell infiltration and microenvironmental remodeling with advancing disease (Figure 3C). Analysis of specific myeloid cell populations revealed an increasing trend in the Z-scores for total macrophages, M1 macrophages, and M2 macrophages from healthy to AD groups, suggesting heightened macrophage infiltration and potential neuroinflammatory activity as AD progresses (Figure 3D). Concurrently, neuronal levels, represented by xCell Z-scores, showed a decreasing trend across the AD continuum (healthy, pre-AD, AD groups). Conversely, scores for pericytes and microvascular endothelial cells exhibited increasing trends, pointing towards simultaneous neurodegeneration and vascular alterations (Figure 3E). Further examination of the relationship between specific cell types and neuropathology revealed significant correlations with Braak stages (Figure 3F). Positive correlations were observed for pericytes (R = 0.36, *p* = 0.0063), M1 macrophages (R = 0.35, *p* = 0.0098), total macrophages (R = 0.33, *p* = 0.012), M2 macrophages (R = 0.29, *p* = 0.033), CD4+ memory T-cells (R = 0.29, *p* = 0.035), and activated dendritic cells (aDC) (R = 0.29, *p* = 0.035). In contrast, neurons exhibited a significant negative correlation with Braak stage (R = −0.46, *p* = 0.00046), reflecting progressive neuronal loss with increasing AD pathology. These findings collectively underscore the complex interplay between neuroinflammation, specific immune cell infiltration patterns, vascular changes, and neuronal degeneration across the Braak stages of Alzheimer’s disease.

### 2.4. Cross-Method Validation Confirms Progressive Macrophage Infiltration, with Emphasis on M2 Polarization, Across Braak Stages of Alzheimer’s Disease

**Figure 4:**
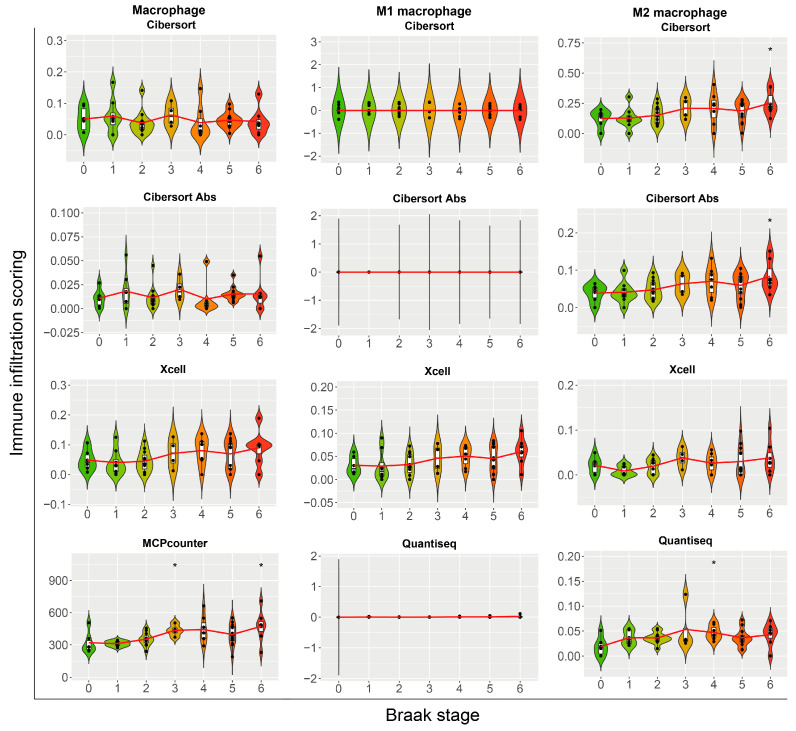
Macrophage Subtype Infiltration Across Braak Stages Estimated by Multiple Deconvolution Methods. Violin plots display the infiltration scores for total macrophages (left), M1 macrophages (middle), and M2 macrophages (right) across Braak stages 0–6. The analysis compares results from four deconvolution methods: CIBERSORT, CIBERSORT Absolute, xCell, and MCPcounter/Quantiseq. In each plot, data points are colored by Braak stage (green to red), a red line indicates the trend, and asterisks denote statistical significance.

To corroborate initial findings, a suite of deconvolution methods, including CIBERSORT, CIBERSORT Absolute, Quantiseq, and MCPcounter, was used to analyze macrophage-related signatures across Braak stages (Figure 4). Analysis of total macrophage scores revealed an upward trend with increasing Braak stage, most clearly detected by MCPcounter. For specific subtype-labeled features, M1 macrophage scores showed a less consistent pattern across methods. In contrast, deconvolution features labeled as M2 macrophages exhibited higher values with advancing Braak stages across CIBERSORT, CIBERSORT Absolute, and Quantiseq. These cross-method results are consistent with progressive enrichment of M2-like myeloid programs during AD neuropathological progression, while acknowledging that these labels represent inferred signatures rather than definitive cell identities.

### 2.5. Independent Validation in GSE33000 Dataset Confirms Broad Macrophage Infiltration in Alzheimer’s Disease, with Multiple Methods Highlighting M2 Subtype Elevation

**Figure 5:**
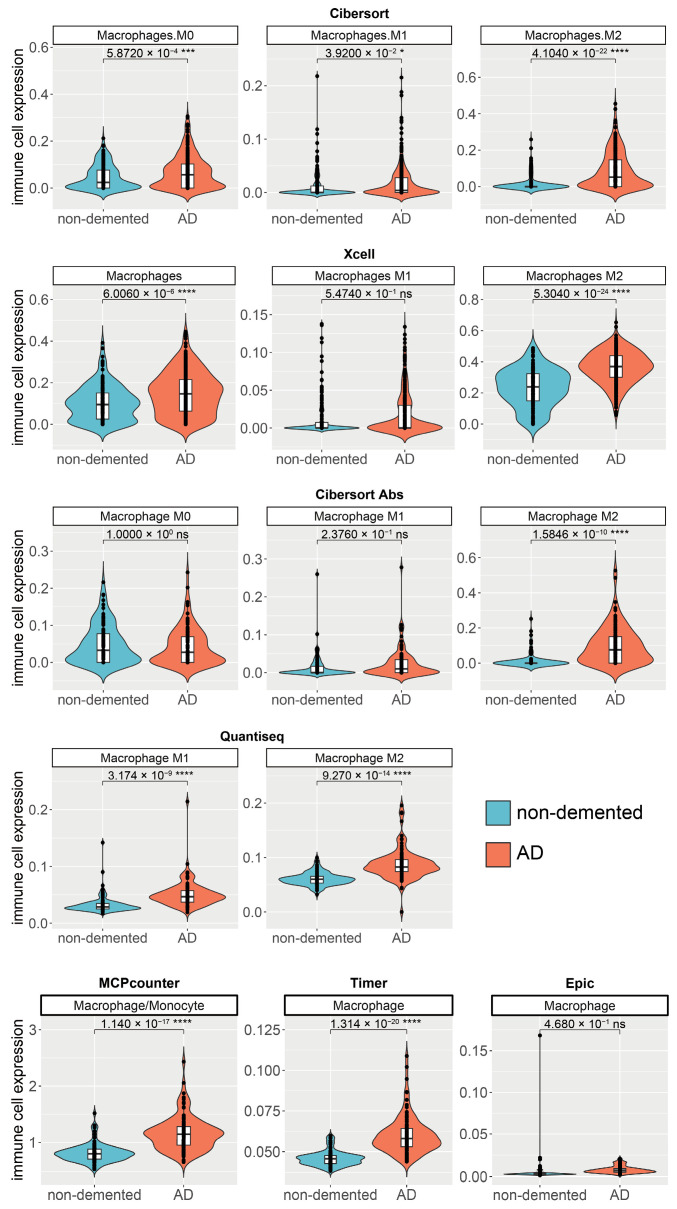
Validation of Differential Macrophage Subtype Infiltration in Alzheimer’s Disease using the GSE33000 Dataset and Multiple Deconvolution Methods. Violin plots illustrate the comparative infiltration levels of various macrophage populations (Macrophages M0, Macrophages M1, Macrophages M2, total Macrophages, or Macrophage/Monocyte, depending on the method) between non-demented controls (blue) and AD patients (red). Immune cell infiltration scores were derived from gene expression data using CIBERSORT, xCell, CIBERSORT Absolute mode (CIBERSORT Abs), quanTIseq, MCPcounter, TIMER, and EPIC. *p*-values for the comparison between non-demented and AD groups are displayed above each plot, with asterisks denoting levels of statistical significance (* *p* < 0.05, *** *p* < 0.001, **** *p* < 0.0001); ‘ns’ indicates no statistical significance.

To validate the initial findings on macrophage infiltration, an independent dataset, GSE33000, was analyzed to compare macrophage presence in AD versus non-demented control samples. Gene expression data from this dataset was interrogated using a comprehensive panel of computational deconvolution methods, including CIBERSORT, xCell, CIBERSORT Absolute mode (CIBERSORT Abs), quanTIseq, MCPcounter, TIMER, and EPIC, to evaluate the infiltration levels of total macrophages (or M0), M1 macrophages, and M2 macrophages (Figure 5). The results consistently demonstrated a significant elevation of macrophage infiltration in AD samples compared to non-demented controls across the majority of methods employed. Specifically, CIBERSORT analysis revealed significant increases in AD patients for Macrophages M0 (*p* = 5.872 × 10^−4^, ***), Macrophages M1 (*p* = 3.920 × 10^−2^, *), and Macrophages M2 (*p* = 4.104 × 10^−22^, ****). xCell analysis corroborated increased infiltration for total Macrophages (*p* = 6.006 × 10^−6^, ****) and Macrophages M2 (*p* = 5.304 × 10^−24^, ****), although Macrophages M1 did not show a significant difference with this method (*p* = 5.474 × 10^−1^, ns). Analysis with CIBERSORT Abs indicated a significant increase only for Macrophages M2 (*p* = 1.584 × 10^−10^, ****) in AD samples. Furthermore, quanTIseq analysis showed significant elevations in AD for both Macrophage M1 (*p* = 3.174 × 10^−9^, ****) and Macrophage M2 (*p* = 9.270 × 10^−14^, ****). MCPcounter (Macrophage/Monocyte, *p* = 1.140 × 10^−17^, ****) and TIMER (Macrophages, *p* = 1.314 × 10^−20^, ****) also demonstrated highly significant increases in macrophage populations in AD. In contrast, the EPIC method did not show a significant difference in macrophage infiltration between AD and non-demented controls (*p* = 4.680 × 10^−1^, ns).

### 2.6. Immune Cell Infiltration Profiles Derived from xCell and CIBERSORT Accurately Predict Alzheimer’s Disease Status and Highlight Key Cellular Contributors

**Figure 6:**
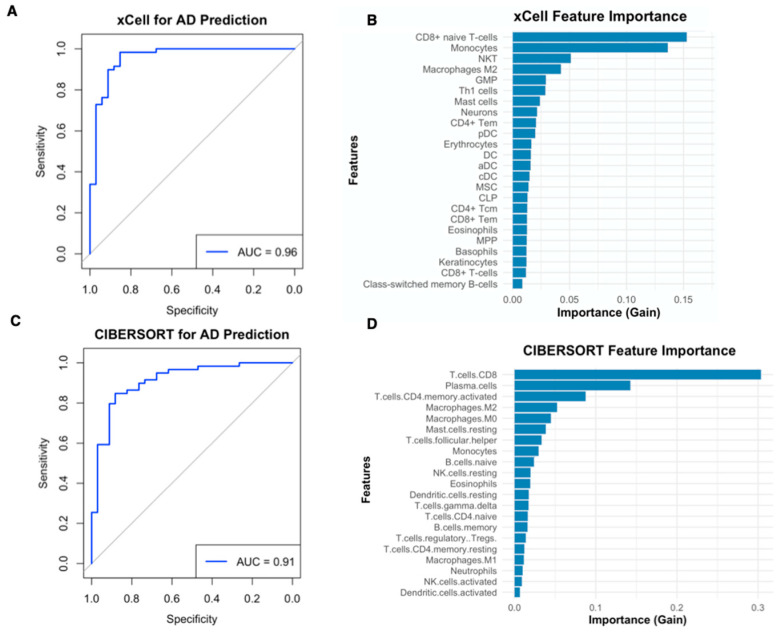
Predictive Performance and Feature Importance of XGBoost Models for Alzheimer’s Disease Classification using Immune Cell Profiles from xCell and CIBERSORT. (**A**) ROC curve for the XGBoost model trained on xCell-derived immune cell enrichment scores from the GSE33000 dataset. (**B**) Feature importance scores for the xCell-based XGBoost model. (**C**) ROC curve for the XGBoost model trained on CIBERSORT-derived immune cell fractions from the GSE33000 dataset. (**D**) Feature importance scores for the CIBERSORT-based XGBoost model. For ROC curves (**A**,**C**), the x-axis represents Specificity and the y-axis represents Sensitivity. For feature importance plots (**B**,**D**), the x-axis represents Importance (Gain) and the y-axis lists the immune cell features.

To evaluate the diagnostic potential of immune cell infiltration profiles in distinguishing AD from non-demented controls, XGBoost machine learning models were trained using immune cell features derived from both CIBERSORT and xCell analyses. Gene expression data from the GSE33000 dataset served as the input for these binary classification models. The model developed using xCell-derived features demonstrated excellent predictive performance, achieving an AUC of 0.96 (Figure 6A). Feature importance analysis for this model identified CD8+ naive T-cells, monocytes, Natural Killer T (NKT) cells, and the deconvolution feature labeled M2 macrophages among the most influential variables for AD classification (Figure 6B). Similarly, the XGBoost model trained with CIBERSORT-derived features also showed strong predictive accuracy, yielding an AUC of 0.91 (Figure 6C). The top contributing features in this model included CD8+ T-cells, plasma cells, and activated CD4+ memory T-cells, while M2 macrophage and M0 macrophage features also carried substantial predictive weight (Figure 6D). Collectively, both modeling approaches demonstrated robust performance in predicting AD status. A consistent finding across both xCell and CIBERSORT methodologies was the emergence of macrophage-related features, particularly the M2-labeled signature, as informative predictors. The superior AUC achieved by the xCell-based model may be attributable to its broader capacity to resolve the cellular heterogeneity and tissue microenvironment components from gene expression data.

### 2.7. CellChat Analyses Suggest Communication-Enriched M2-like Myeloid Interaction Niches in the Alzheimer’s Disease Microenvironment

**Figure 7:**
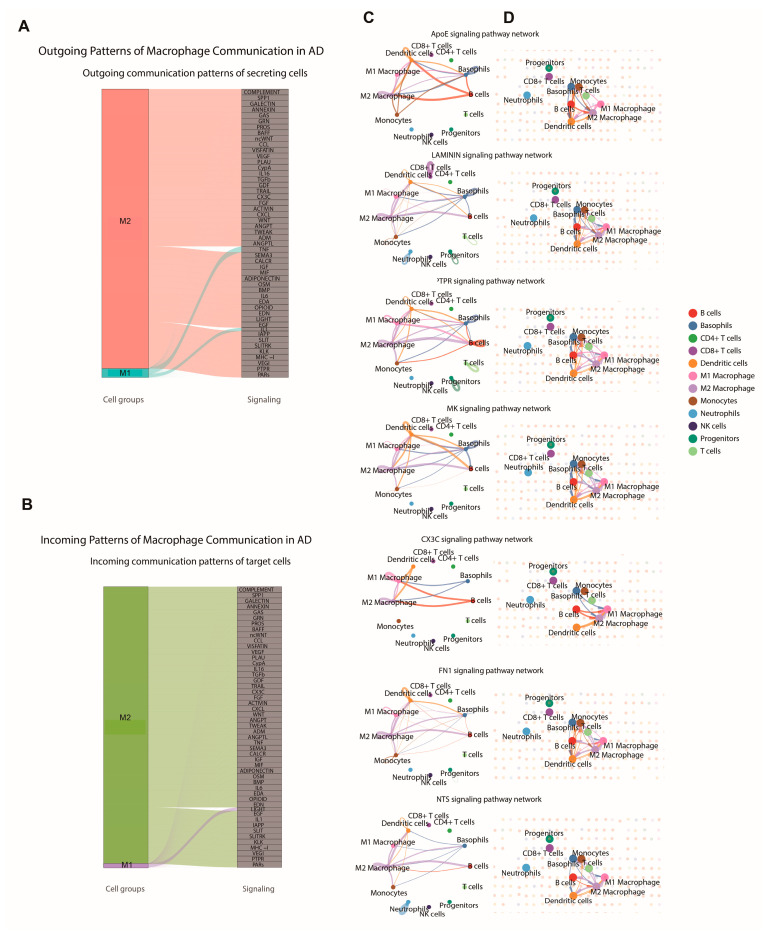
CellChat Analyses Suggest Communication-Enriched M2-Like Myeloid Interaction Networks in Alzheimer’s Disease. (**A**) River plot illustrating inferred outgoing signaling patterns from operationally defined M1-like and M2-like myeloid populations in AD samples based on human single-cell RNA sequencing (RNA-seq) data. In (**A**,**B**), colored blocks indicate M1-like and M2-like myeloid groups; stream colors denote signaling flows, and width reflects inferred communication probability. (**B**) River plot showing inferred incoming signaling patterns received by operationally defined M1-like and M2-like myeloid populations in AD samples derived from human single-cell RNA-seq data. (**C**) Circle plots visualizing inferred intercellular communication networks for seven representative signaling pathways (ApoE, LAMININ, PTPR, MK, CX3C, *FN1*, NTS) derived from spatial transcriptomic data from an AD mouse model. Each node represents a distinct immune or stromal cell type. The thickness of the edges reflects the relative strength of inferred interactions between sender and receiver cell types for the specified pathway. (**D**) Spatial CellChat plots.

To investigate whether macrophage and myeloid activation states were associated with distinct communication patterns in AD, cell-cell communication was inferred using the CellChat algorithm. In the human single-cell RNA-seq dataset (GSE146639), outgoing and incoming signaling patterns were compared between operationally defined M1-like and M2-like myeloid signatures. M2-like cells showed broader outgoing pathway participation (Figure 7A), including VEGF, TGFβ, CXCL, and ACTIVIN-associated signals, whereas M1-like cells contributed to fewer outgoing pathways. A similar trend was observed for incoming signals (Figure 7B), with M2-like cells receiving a wider range of inferred inputs, including *IL6*, *TNF*, WNT, and SLITRK-associated signaling. These findings are consistent with an interaction-enriched position for M2-like myeloid signatures within the inferred AD communication network. We next examined whether comparable interaction patterns could be spatially contextualized using exploratory spatial transcriptomic data from an AD mouse model. In this setting, M2-like myeloid signatures participated in inferred signaling relationships with monocytes, B cells, dendritic cells, and M1-like myeloid populations. Representative pathways included *ApoE*, CX3C, *FN1*, LAMININ, MK, NTS, and PTPR (Figure 7C). For example, *ApoE*-associated signaling connected M2-like myeloid signatures with monocytes and B cells, whereas CX3C and *FN1*-associated interactions were prominent with dendritic cells. Spatial CellChat analysis further suggested that these interactions were enriched in localized niches where M2-like myeloid, dendritic, B-cell, monocyte, and M1-like signals co-occurred in close proximity (Figure 7D). Because these spatial analyses were performed in mouse tissue, they should be interpreted as exploratory contextual support rather than direct evidence of human-equivalent spatial mechanisms.

## 3. Discussion

Our integrated transcriptomic analyses suggest that M2-like macrophage and myeloid signatures are progressively enriched with AD neuropathological severity and are computationally inferred to participate in extensive intercellular communication networks. Across bulk deconvolution, pathway enrichment, machine-learning feature ranking, single-cell communication analysis, and spatial contextualization, these signatures were consistently associated with AD-related neuroimmune remodeling. We therefore interpret M2-like myeloid programs as hypothesis-generating candidate contributors to the AD immune microenvironment rather than established causal drivers of disease progression. This more conservative interpretation is important because the present evidence is correlative and computational, not interventional.

An important implication of our findings is that the M1/M2 framework should be interpreted cautiously. Macrophage and microglial activation states exist along a dynamic spectrum, and transcriptomic signatures assigned as M1-like or M2-like may capture overlapping, transitional, or context-dependent programs rather than discrete functional subtypes [29,30,31,32,33,34]. In this study, the concurrent increase in M1-like and M2-like features with AD severity is therefore better understood as evidence of a complex dysregulated myeloid environment than as a binary phenotypic switch. The enrichment of M2-like programs may reflect reparative, immunosuppressive, fibrotic-like, or maladaptive responses that coexist with persistent inflammatory signaling, depending on disease stage and microenvironmental context.

Within this framework, the association of M2-like myeloid signatures with broad inferred ligand-receptor connectivity remains biologically informative. In the human single-cell dataset, M2-like cells displayed wider outgoing and incoming CellChat pathway participation than M1-like cells, and in the exploratory mouse spatial dataset these signals were localized to interaction-enriched niches involving monocytes, dendritic cells, B cells, and other myeloid populations. These networks included *ApoE*, CX3C, *FN1*, LAMININ, MK, NTS, and PTPR-associated signaling contexts, which are relevant to lipid handling, chemotaxis, matrix remodeling, and neuroimmune regulation [35,36,37,38,39,40]. Likewise, macrophage-related features ranked highly in XGBoost classifiers that distinguished AD from controls. Together, these observations suggest that M2-like myeloid programs track with AD-associated immune remodeling and may serve as informative biomarkers or network-level readouts, while still requiring orthogonal validation to establish their cellular identity and functional consequence.

Biochemical implications of M2-like myeloid remodeling in AD also merit consideration. Although our transcriptomic data cannot directly measure redox flux, mitochondrial respiration, or lipid peroxidation, the enrichment of nitric oxide-, TGF-β-, and ferroptosis-related pathways suggests that M2-like myeloid remodeling may intersect with biochemical processes known to influence AD neurotoxicity [13,19,20,41,42]. TGF-β-associated programs may reflect tissue repair, immune suppression, or fibrotic-like remodeling, whereas nitric oxide signaling can participate in neurovascular regulation but also contribute to nitrosative stress when dysregulated. Likewise, ferroptosis-related signatures point to possible interactions among iron handling, lipid peroxidation, mitochondrial dysfunction, and chronic inflammatory activation. In this setting, long-standing myeloid responses that are initially adaptive may become maladaptive if oxidative and bioenergetic stress remain unresolved.

Metal dyshomeostasis represents an additional biochemical axis that was not directly interrogated in the present study. Iron accumulation can amplify oxidative stress, lipid peroxidation, and ferroptosis, potentially intersecting with the myeloid programs identified here [42,43]. Copper and zinc may also influence amyloid aggregation, synaptic function, oxidative stress, and immune signaling, raising the possibility that metal-dependent biochemical stress both shapes and is shaped by AD-associated myeloid remodeling [43]. Future studies integrating transcriptomics with metallomics, iron-sensitive imaging, histochemical staining, spatial proteomics, or ICP-MS-based measurements will be required to determine whether metal-dependent pathology contributes to the inferred communication states described in this work.

### Limitations

Several methodological limitations warrant explicit emphasis. First, the study is cross-sectional and entirely in silico; therefore, association with Braak stage or AD status should not be interpreted as evidence that M2-like myeloid programs drive disease progression. Second, bulk transcriptomic deconvolution cannot cleanly distinguish resident microglia, infiltrating monocyte-derived macrophages, and perivascular macrophages, so the inferred M2 signal should be interpreted as M2-like myeloid enrichment rather than a definitive cellular subtype. Third, the M1 and M2 labels used in both deconvolution and single-cell or spatial analyses are operational transcriptomic categories imposed on a biological continuum, and intermediate or mixed activation states may be misclassified. Fourth, the spatial validation layer relied on a mouse AD model because human spatial datasets with sufficient immune-cell resolution remain limited; inter-species differences in tissue architecture and myeloid biology constrain direct extrapolation to human AD. Fifth, the inferred communication networks, pathway enrichments, and machine-learning rankings are algorithm-dependent and lack direct protein-level or functional validation. Finally, the present study did not directly measure redox balance, mitochondrial function, ferroptotic flux, or metal ion concentrations, leaving the proposed biochemical links as testable hypotheses. Future work should integrate longitudinal human cohorts with immunohistochemistry, spatial proteomics, cytokine profiling, phagocytosis or metabolic assays, and metallomic measurements to validate these computational inferences.

## 4. Materials and Methods

### 4.1. Dataset Acquisition and Preprocessing

Gene expression data were sourced from the NCBI Gene Expression Omnibus (GEO; National Center for Biotechnology Information, Bethesda, MD, USA) database. The first dataset, GSE106241, comprised gene expression profiles from human inferior temporal cortex samples across Braak stages of Alzheimer’s disease (AD) progression [44]. Using GEOquery (version 2.76.0) in R (version 4.5.1), data were downloaded and mapped to gene symbols via platform annotation GPL24170. Probes lacking annotations and duplicates were removed, yielding a final matrix of unique genes. Phenotype data (Braak stage, age, gender, etc.) were curated, and samples with missing information excluded. The dataset included 60 samples: 6 Healthy (Braak 0), 28 pre-AD (Braak 1–3), and 26 AD (Braak 4–6). A second dataset, GSE33000, was also processed to validate findings [45]. It contains prefrontal cortex profiles from 467 samples (310 AD, 157 controls), annotated using GPL4372. Similar preprocessing ensured data integrity.

### 4.2. Gene Set Variation Analysis (GSVA)

GSVA was performed on the normalized gene expression data from the processed GSE106241 dataset to assess pathway activity changes on a per-sample basis [46]. The gene sets for this analysis were obtained from the Molecular Signatures Database (MSigDB) [47]. The GSVA was conducted using the GSVA package (version 2.2.1) in R. Enrichment scores were computed using the gsva() function, with parameters set to method = “gsva”, kcdf = “Gaussian,” and min.sz = 2 to include gene sets containing at least two genes. Following the calculation of GSVA enrichment scores for each pathway per sample, Pearson correlation coefficients were computed between these scores and the corresponding Braak stages of the samples. Pathways demonstrating statistically significant correlations with AD progression (*p*-value < 0.05) were identified. For subsequent focused analysis and visualization, the top five positively and top five negatively correlated gene sets with Braak stage were highlighted for each interrogated MSigDB database category.

### 4.3. Weighted Gene Co-Expression Network Analysis (WGCNA)

WGCNA was performed to identify co-expressed pathway modules based on GSVA scores from the GSE106241 dataset [48]. Using the WGCNA R package (version 1.74), the GSVA matrix was transposed to set samples as rows and pathways as columns. Quality control via goodSamplesGenes() excluded pathways or samples with missing values. Hierarchical clustering (average linkage, Euclidean distance) and cutreeStatic() were applied to remove outliers. The soft-thresholding power (β) was determined with pickSoftThreshold(), selecting β = 6 to achieve a scale-free topology fit (R^2^ > 0.9). An unsigned co-expression network was constructed using blockwiseModules() (power = 6, minModuleSize = 30, mergeCutHeight = 0.2, TOMType = “unsigned”), identifying modules via dynamic tree cutting and merging those with similar eigengenes. Module eigengenes (MEs), representing the first principal component of each module, were calculated and correlated with clinical traits such as Braak stage using Pearson correlation (cor()), and significance assessed with corPvalueStudent().

### 4.4. Immune Cell Infiltration Analysis

To estimate immune cell proportions in brain tissue samples from GSE106241 and GSE33000, analyses were performed using the IOBR R package (version 0.99.0) [49], which integrates multiple deconvolution methods. xCell was applied via its web tool to compute enrichment scores for 64 immune and stromal cell types [50], while CIBERSORT estimated the relative fractions of 22 predefined immune cell types [51]. Additional tools, including TIMER [52], EPIC [53], quanTIseq [54], and MCPcounter [55], provided complementary perspectives on immune infiltration. Abundance estimates from all methods were combined for comparative analysis. Data were reshaped into long format with tidyr (version 1.3.2) and analyzed using rstatix (version 0.7.3). Independent *t*-tests compared immune cell proportions between AD and controls, with Holm-Bonferroni correction applied to *p*-values. Visualizations were created with ggplot2 (version 4.0.2) and ggpubr (version 0.6.3), including violin plots overlaid with boxplots and jittered points, annotated with adjusted *p*-values via stat_*p*value_manual().

### 4.5. XGBoost Model Training and Feature Importance Analysis

The input features for XGBoost models (version 3.2.1.1), representing immune cell compositions, were derived from GSE33000 gene expression data using two deconvolution methods: CIBERSORT and xCell. Immune cell abundance matrices were generated separately and preprocessed by removing sample identifiers and normalizing feature values. Phenotype labels were defined as ‘1’ for AD and ‘0’ for non-demented controls. Each feature set was split into training (80%) and testing (20%) sets using stratified sampling to preserve class proportions. Feature matrices and labels were converted into xgb.DMatrix objects optimized for XGBoost [56]. Separate models for CIBERSORT and xCell were trained with 100 boosting rounds, max_depth = 6, eta = 0.3, and binary:logistic as the objective function. Class imbalance was addressed by adjusting scale_pos_weight to the control/AD ratio. Model performance was evaluated on testing sets using accuracy and AUC metrics. ROC curves were plotted with pROC, and feature importance scores were extracted via xgb.importance() and visualized with ggplot2.

### 4.6. Single-Cell RNA Sequencing (RNA-Seq) Processing and Cell-Cell Communication Analysis

The single-cell RNA sequencing dataset GSE146639 was obtained from NCBI GEO [57,58], comprising transcriptomic profiles of human microglia from post-mortem cortical brain tissue of 27 donors clinically classified as controls (CTR), controls with amyloid and tau pathology (CTR+), and Alzheimer’s disease (AD) patients. Raw count matrices were downloaded, aligned to a unified gene list, and merged. We used the processed cell matrix provided by the study and applied additional filtering by removing genes with >90% missing values; Ensembl IDs were mapped to HGNC symbols using biomaRt (version 2.64.0), and genes lacking valid HGNC symbols were excluded. The expression matrix was log-normalized, and metadata were used to retain donor-group annotations for downstream analyses. Initial cell identities were assigned with SingleR (version 2.10.0) using HumanPrimaryCellAtlasData as reference. To evaluate polarization-associated programs, cells with myeloid and microglial annotations were scored using canonical M1-like (e.g., *TNF*, *IL6*, *IL1B*) and M2-like (e.g., *CD163*, *MRC1*, *IL10*, *TGFB1*) marker sets curated from established macrophage polarization literature [29,30]. Cells were then operationally annotated as M1-like or M2-like on the basis of relative marker expression. Because macrophage and microglial activation states exist along a continuum, these labels were used as operational transcriptomic signatures rather than discrete functional phenotypes or evidence of peripheral macrophage identity. CellChat (version 2.2.0) was subsequently used to analyze group-specific cell-cell communication networks, focusing on “Secreted Signaling” pathways. Overexpressed genes and significant interactions were identified, communication probabilities were computed, and non-negative matrix factorization (NMF) was used to summarize outgoing and incoming signaling patterns for the M1-like and M2-like populations.

### 4.7. Spatial Transcriptomics Data Acquisition and Preprocessing

To investigate whether the inferred myeloid communication patterns could be spatially contextualized, a spatial transcriptomics dataset was obtained from ssREAD (https://bmblx.bmi.osumc.edu/ssread/; accessed on 14 October 2025). Because spatially resolved human AD datasets with sufficient immune-cell resolution remain limited, mouse brain data from Navarro and Croteau et al., including hippocampal and olfactory bulb tissue sections from wild-type and AD model mice, were used as an exploratory spatial validation layer rather than a direct human-equivalent mechanistic dataset [41]. Raw count matrices (.tsv files) were processed with data.table in R, filtered, aligned to common genes, and merged. Spatial coordinates derived from spot identifiers were linked to genotype metadata. A Seurat object was created using SCTransform normalization followed by PCA/UMAP and graph-based clustering. Cell identities were annotated with SingleR using Monaco Immune Data, with emphasis on microglia and macrophage-enriched spots. These myeloid-enriched spots were then evaluated for operational M1-like (e.g., *Cd86*, *H2-Ab1*) and M2-like (e.g., *Mrc1*, *Tgfb1*) programs. As in the single-cell analysis, these labels were interpreted as activation-state signatures rather than discrete binary cell types. CellChat was applied using CellChatDB.mouse to compute proximity-weighted signaling probabilities and to visualize representative ligand-receptor pathways with netVisual_aggregate. These spatial analyses were intended to examine whether macrophage and myeloid communication patterns inferred from human data could be observed in an AD-like tissue environment, not to establish direct human spatial mechanisms.

## 5. Conclusions

Through an integrated multi-omics framework spanning bulk transcriptomics, single-cell profiling, and exploratory spatial analyses, this study shows that M2-like macrophage and myeloid signatures are consistently associated with Braak stage, AD status, and inferred immune communication remodeling. These signatures ranked among the more informative immune features in machine-learning models and occupied communication-enriched positions in CellChat analyses, including *ApoE*, CX3C, and *FN1*-associated signaling contexts. Rather than establishing these programs as disease drivers, our findings support a model in which M2-like myeloid programs represent candidate components of the AD neuroimmune milieu whose functions are likely context-dependent and intertwined with broader inflammatory, metabolic, and tissue-remodeling processes. Future studies in human-relevant experimental systems will be necessary to resolve cell identity, validate protein-level and functional effects, and determine whether modulating these myeloid programs has therapeutic value in Alzheimer’s disease.

## Data Availability

All datasets analyzed in this study are publicly available. Bulk RNA-seq and single-cell RNA-seq data were obtained from the GEO under accession numbers GSE33000, GSE106241, and GSE146639, while spatial transcriptomic data were retrieved from ssREAD [43]. Processed data matrices, cell-type annotations, and scripts used for integrative analyses are available upon reasonable request from the corresponding author. No proprietary or restricted-access datasets were used in this study. During manuscript preparation, ChatGPT (OpenAI, model o3) was used only for language editing and stylistic refinement. The authors provided all scientific concepts, data analyses, interpretations, and conclusions. All AI-assisted suggestions were critically reviewed, revised where necessary, and approved by the authors, who take full responsibility for the final content.

## Abbreviations

The following abbreviations are used in this manuscript:

AD	Alzheimer’s disease
AUC	Area Under Curve
aDC	Activated Dendritic Cell
APOE	Apolipoprotein E
APP	Amyloid Precursor Protein
CIBERSORT	Cell-type Identification By Estimating Relative Subsets Of RNA Transcripts
DAM	Disease-Associated Microglia
EPIC	Estimating the Proportions of Immune and Cancer cells
GEO	Gene Expression Omnibus
GO	Gene Ontology
GOCC	Gene Ontology Cellular Component
GOBP	Gene Ontology Biological Process
GOMF	Gene Ontology Molecular Function
GSVA	Gene Set Variation Analysis
HGNC	Human Genome Organisation Gene Nomenclature Committee
IOBR	Immuno-Oncology Biological Research
LPS	Lipopolysaccharide
MCPcounter	Microenvironment Cell Populations counter
ME	Module Eigengene
MSigDB	Molecular Signatures Database
NCBI	National Center for Biotechnology Information
NKT	Natural Killer T (cell)
PCA	Principal Component Analysis
PSEN1	Presenilin 1
PSEN2	Presenilin 2
ROC	Receiver Operating Characteristic curve
scRNA-seq	Single-cell RNA sequencing
TIMER	Tumor Immune Estimation Resource
TNF	Tumor Necrosis Factor
TREM2	Triggering Receptor Expressed on Myeloid Cells 2
UMAP	Uniform Manifold Approximation and Projection
VEGF	Vascular Endothelial Growth Factor
WGCNA	Weighted Gene Co-expression Network Analysis
XGBoost	eXtreme Gradient Boosting
